# The effect of preoperative stellate ganglion block on postoperative delirium in elderly patients undergoing gastrointestinal oncologic surgery: protocol for a randomized, double-blind, sham-controlled trial

**DOI:** 10.3389/fneur.2026.1804153

**Published:** 2026-06-16

**Authors:** Rongrong Song, Yuqin Huang, Jie Liu, Xingyu Wang, Yuqian Liu, Fanli Wang, Fang Wu, XiaXia Li, Yang Liu, Lu Cao, Yan Liu, Guojieying Wang, Minghong Gui, Yingbin Wang, Xiangfei Huang

**Affiliations:** 1 Department of Anesthesiology, The Second Hospital & Clinical Medical School, Lanzhou University, Lanzhou, China; 2 Department of Tumor Surgery and General Surgery, Gansu Provincial Key Laboratory of Gastrointestinal Cancer, The Second Hospital & Clinical Medical School, Lanzhou University, Lanzhou, China; 3 Key Laboratory of Environmental Oncology in Gansu Province, The Second Hospital & Clinical Medical School, Lanzhou University, Lanzhou, China; 4 Center for Digestive System Tumor Transformation and Innovation Engineering, Environmental Oncology Center, Lanzhou University, Lanzhou, China; 5 Department of Operations Management, The Second Hospital & Clinical Medical School, Lanzhou University, Lanzhou, China

**Keywords:** elderly, gastrointestinal oncologic surgery, postoperative delirium, randomized controlled trial, stellate ganglion block

## Abstract

**Background:**

Postoperative delirium (POD) is a common and severe complication in elderly patients following gastrointestinal oncologic surgery, associated with prolonged hospitalization, increased morbidity and mortality, and substantial healthcare costs. The stellate ganglion block (SGB), a sympathetic nerve block with anti-inflammatory and autonomic-modulating effects, has shown promise in improving perioperative outcomes. However, high-quality randomized controlled trials evaluating its efficacy in preventing POD in this older population are lacking.

**Methods:**

This is a single-center, prospective, randomized, double-blind (participants and outcome assessors), sham- controlled trial. A total of 174 elderly patients (≥60 years) scheduled for elective radical gastrointestinal cancer surgery will be randomized in a 1:1 ratio to receive either two ultrasound-guided SGBs (one on the afternoon before surgery and one 30 min before anesthesia induction) with 0.375% ropivacaine (3–5 mL each) or a sham block with normal saline (3–5 mL). All patients, surgeons, and outcome assessors will be blinded to group assignment. A standardized anesthesia protocol and a multicomponent delirium- prevention bundle will be applied to all participants. The primary outcome is the cumulative incidence of POD within postoperative days 1–3, assessed twice daily using the 3-Minute Diagnostic Interview for CAM-defined Delirium (3D-CAM). Secondary outcomes include delirium severity and duration, postoperative recovery (Quality of Recovery-15 score), inflammatory biomarkers (procalcitonin, C-reactive protein (CRP), interleukin-6 (IL-6), lymphocyte-to-monocyte ratio [LMR], neutrophil-to-lymphocyte ratio [NLR]), pain trajectories, opioid consumption, and length of hospital stay.

**Expected outcomes:**

We hypothesize that preoperative SGB will reduce the incidence and severity of POD compared to a sham block. This study will provide preliminary evidence on the feasibility and efficacy of SGB as a targeted intervention for high-risk older surgical oncology patients, while acknowledging that the assumed effect size may be optimistic.

**Clinical trial registration:**

https://www.chictr.org.cn/showproj.html?proj=302103, identifier: ChiCTR2600118223.

## 1 Introduction

### 1.1 Background and rationale

Postoperative delirium (POD) is an acute, fluctuating neuropsychiatric syndrome characterized by disturbances in attention, consciousness, and cognition (1). Among elderly patients (≥60 years) undergoing surgery for gastrointestinal malignancies, POD occurs in 35%~48% of cases and is consistently associated with poor outcomes, including increased risk of postoperative complications, prolonged hospitalization, institutionalization, long-term cognitive decline, and mortality (2–4). Despite these profound consequences, effective pharmacological prevention strategies remain limited, particularly in the geriatric surgical population (5).

Current clinical practice primarily relies on non-pharmacologic interventions such as multicomponent prevention bundles (e.g., the ABCDEF approach) (6), yet these strategies alone cannot fully mitigate POD risk in older adults. Given the multifactorial pathophysiology of POD, including neuro-inflammation (7), autonomic dysregulation (8), and impaired cerebral perfusion (9), novel strategies targeting neuro-inflammation and sympathetic over-activity are needed, especially for elderly patients at higher risk.

### 1.2 Stellate ganglion block as a promising intervention

The stellate ganglion is a key sympathetic structure. Ultrasound-guided stellate ganglion block (SGB) is a minimally invasive procedure that transiently inhibits sympathetic outflow (10). Preclinical and emerging clinical evidence suggests that SGB may confer neuroprotective benefits by: (1) modulating systemic and neuro-inflammation through cytokine regulation (11); (2) improving cerebral blood flow via vasodilation (12); (3) attenuating perioperative stress and hyper-adrenergic states (13). These mechanisms align closely with proposed pathways of POD pathogenesis. Recent studies have reported that SGB may reduce postoperative pain, inflammatory markers, and incidence of postoperative cognitive dysfunction (POCD) in animal models (14, 15). Meanwhile, it is important to note that these studies focused on POCD and other perioperative outcomes, not directly on POD. Direct evidence for SGB preventing POD in humans remains limited, and the available studies are small in sample size or still at the protocol stage. However, there is a paucity of rigorous, blinded, randomized controlled trials specifically designed to evaluate the efficacy of SGB for preventing POD in elderly patients undergoing major gastrointestinal oncologic surgery (16, 17). This study aims to address this evidence gap.

## 2 Study objectives

### 2.1 Primary objective

To compare the effect of preoperative ultrasound-guided SGB versus sham block on the 3-day cumulative incidence of POD, following a protocol of two blocks: the first administered on the afternoon before surgery, and the second administered 30 min before anesthesia induction.

### 2.2 Secondary objectives

To evaluate the effects of SGB on delirium severity and duration, postoperative recovery (Quality of Recovery-15 score), inflammatory biomarkers (procalcitonin, C-reactive protein (CRP), interleukin-6 (IL-6), lymphocyte-to-monocyte ratio [LMR], neutrophil-to-lymphocyte ratio [NLR]), pain trajectories, opioid consumption, and length of hospital stay.

### 2.3 Hypothesis

We hypothesize that patients receiving preoperative SGB will exhibit a significantly lower incidence and severity of POD, attributable to its anti-inflammatory and autonomic-modulating properties.

## 3 Methods

### 3.1 Study design and setting

This is a prospective, randomized, double-blind, sham-controlled, single-center trial conducted at t the Second Hospital & Clinical Medical School, Lanzhou University. The protocol adheres to the SPIRIT 2025 and CONSORT 2025 guidelines.

#### 3.1.1 Trial registration and recruitment status

This study was registered with the Chinese Clinical Trial Registry (ChiCTR) before the enrollment of the first participant (Registration No. ChiCTR2600118223). As of the time of manuscript submission, no participants have been enrolled or randomized. Recruitment is planned to take place from February 5, 2026, to March 1, 2027.

### 3.2 Participants

**Figure 1:**
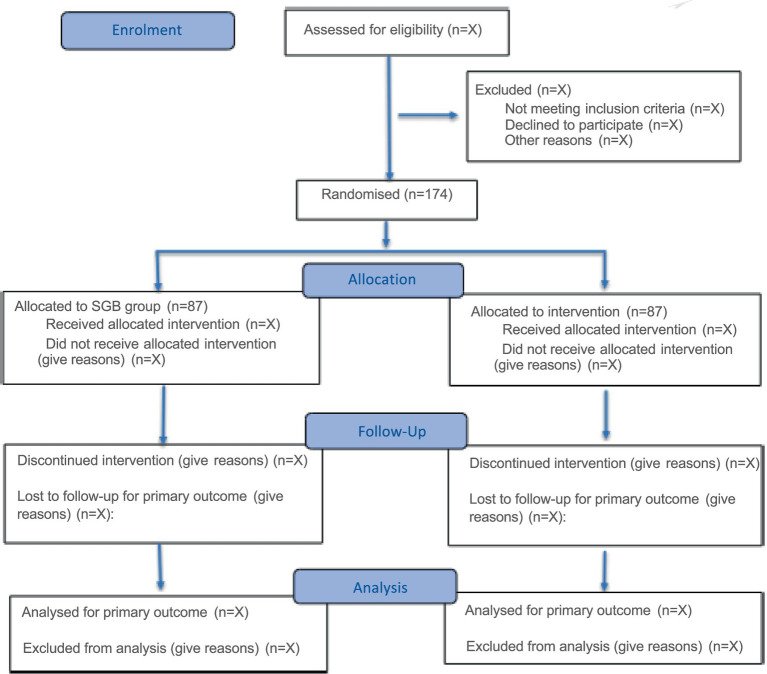
CONSORT 2025 flow diagram (22).

The participant enrollment and study workflow are detailed in Figure 1.

#### 3.2.1 Inclusion criteria

(1) Age ≥ 60 years.
(2) Scheduled for elective open or laparoscopic radical resection of gastric or colorectal cancer.
(3) American Society of Anesthesiologists (ASA) physical status I-III.
(4) Ability to provide written informed consent (or through a legal representative).

#### 3.2.2 Exclusion criteria

(1) Preexisting cognitive impairment (Montreal Cognitive Assessment score below education-adjusted cutoff) or diagnosed dementia.
(2) Severe visual/hearing impairment or language barrier precluding reliable delirium assessment.
(3) Major psychiatric disorders (e.g., schizophrenia, bipolar disorder).
(4) Contraindications to SGB: local infection at the puncture site, known allergy to local anesthetics, severe coagulopathy, or therapeutic anticoagulation that cannot be safely paused.
(5) Severe systemic disease: Child-Pugh C liver cirrhosis, end-stage renal disease (eGFR <30 mL/min/1.73 m^2^ or on dialysis), NYHA class III-IV heart failure.
(6) Emergency surgery, anticipated surgery duration <2 h or >6 h.
(7) History of substance abuse.

### 3.3 Randomization and blinding

Participants will be randomized in a 1:1 ratio to the SGB or sham group using computer- generated, permuted block randomization (block sizes of 4 and 6). Allocation will be concealed using sequentially numbered, opaque, sealed envelopes (SNOSE) prepared by an independent statistician. The envelope will be opened only by the research nurse preparing the study syringe.

This study is double-blind with respect to participants and outcome assessors. Participants, surgeons, postoperative care teams, and outcome assessors will be unaware of group allocation. The anesthesiologist performing the block will not be blinded due to the potential physical signs of a successful block (e.g., Horner’s syndrome) but will have no role in postoperative assessments or data collection.

Although successful SGB may produce visible signs such as Horner’s syndrome (ptosis, miosis, anhidrosis), these signs are typically ipsilateral and may be noticeable to the participant and potentially to the assessor. To evaluate the success of blinding, the following measures will be implemented:

(1) Participant blinding assessment: Within 24 h after the final postoperative delirium assessment, participants will be asked to guess their group assignment (SGB/sham/uncertain). The proportion of correct guesses will be compared between groups using a chi-square test to assess whether blinding was maintained.
(2) Assessor blinding assessment: Outcome assessors will be asked to document any suspicion of group allocation (and the reason, e.g., observed Horner’s syndrome) after completing each participant’s delirium assessments. The frequency of suspected unblinding will be reported.
(3) Mitigation strategies: To minimize unblinding, assessors will be instructed to avoid direct eye contact focused on the participant’s pupillary status and to focus on standardized assessment procedures. Participants will be instructed not to discuss any periprocedural sensations (e.g., facial warmth, ptosis) with assessors.

Results of the blinding effectiveness assessment will be reported alongside the primary outcome as a secondary analysis.

Unblinding will be permissible only in the event of a serious adverse event where knowledge of the intervention is crucial for clinical management. The unblinding procedure will be overseen by the principal investigator.

### 3.4 Interventions

#### 3.4.1 Active intervention (SGB group)

Patients in this group will receive two stellate ganglion blocks: The first block will be performed on the afternoon before surgery. The second will be performed 30 min before the induction of general anesthesia on the day of surgery.

Lateralization: All blocks will be performed on the right side at the C6 level. The right side is selected for the following reasons: (1) right-sided SGB is associated with a lower risk of esophageal injury and has a more predictable anatomical relationship between the stellate ganglion and adjacent structures; (2) right-sided sympathetic blockade may have a more favorable effect on cardiac autonomic balance (reducing sympathetic outflow to the heart) compared to left-sided block; (3) previous pilot studies on SGB for perioperative neurocognitive outcomes predominantly used right-sided approach, facilitating comparison with existing literature.

**Figure 2:**
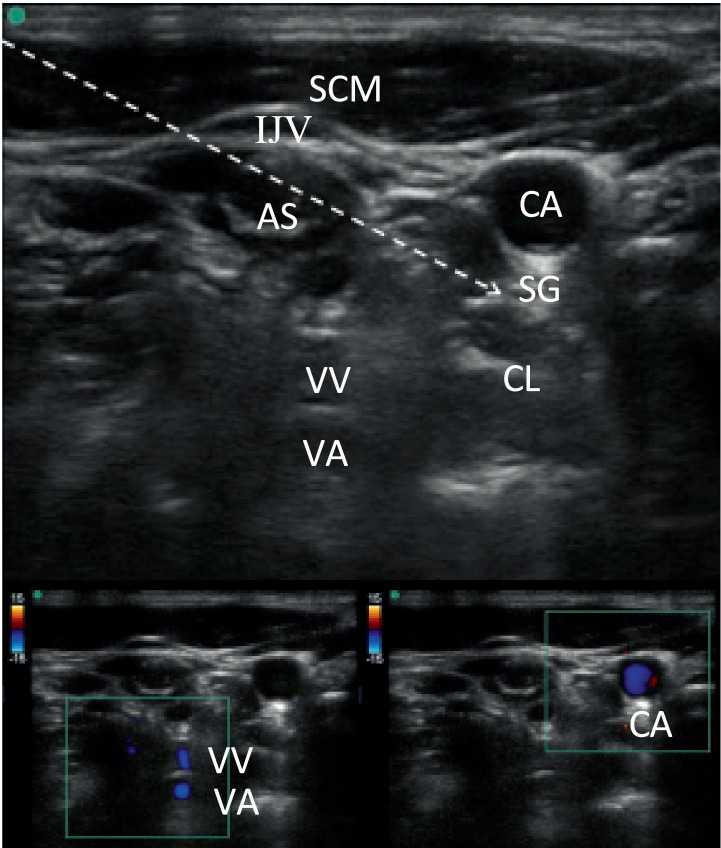
Ultrasound-guided stellate ganglion block. SCM, sternocleidomastoid muscle; IJV, internal jugular vein; CA, carotid artery; AS, anterior scalene; VV, vertebral vein; VA, vertebral artery; SG, stellate ganglion; LCM, longus colli muscle.

Under sterile conditions and real-time ultrasound guidance (C6 level), a 22-gage needle will be advanced to the prevertebral fascia. After negative aspiration, 3–5 mL of 0.375% ropivacaine will be injected (Figure 2). Successful blockade will be confirmed by the development of Horner’s syndrome (ptosis, miosis, anhidrosis) and/or ipsilateral skin temperature increase ≥1.5 °C within 15 min.

#### 3.4.2 Sham intervention (control group)

An identical procedure will be performed at the corresponding time points (on the afternoon before surgery and 30 min before anesthesia induction on the surgery day), including skin disinfection, ultrasound imaging, and needle placement. The syringe will contain 3–5 mL of normal saline (0.9% NaCl). No local anesthetic will be administered. The rationale for using normal saline as the sham control is provided in section 3.4.3.

#### 3.4.3 Rationale for the sham control

This study uses periganglionic normal saline injection as the sham control. The rationale for this design is based on the following considerations:

##### 3.4.3.1 Procedural matching and blinding maintenance

Normal saline injection is highly consistent with active SGB in terms of needle trajectory, ultrasound imaging, procedural tactile sensation, and postoperative subjective experience. This ensures effective blinding of participants and outcome assessors, minimizing assessment bias that could arise from procedural differences.

##### 3.4.3.2 Absence of specific sympathetic blockade effect

Normal saline lacks the nerve conduction blocking effect of local anesthetics and therefore does not produce the specific sympathetic inhibitory effects of SGB (e.g., ipsilateral skin temperature increase, Horner’s syndrome). It has no specific therapeutic effect, consistent with the basic definition of a sham control.

##### 3.4.3.3 Controllability of non-specific effects

Although the injection procedure may produce minor mechanical stimulation or placebo effects, such non-specific effects are balanced between the two groups and are substantially smaller than the specific SGB effects mediated by local anesthetics. Several previous randomized controlled trials of SGB have used normal saline injection as a sham control, confirming its effectiveness in maintaining blinding and controlling bias (13).

Therefore, the normal saline sham control design used in this study balances the two key principles of blinding maintenance and absence of specific intervention effects, representing a scientifically valid and literature-supported approach.

### 3.5 Safety management

#### 3.5.1 Operator qualifications

All SGB procedures in this study will be performed by anesthesiologists with at least 3 years of experience in ultrasound-guided nerve blocks. Each operator must complete a dedicated SGB training program and pass an institutional competency assessment, demonstrating the ability to identify vascular variations, adjust the needle trajectory, and manage potential complications independently.

#### 3.5.2 Safety measures during the procedure

(1) Pre-procedural assessment: All patients will undergo routine coagulation testing (PT, APTT, INR, platelet count) before the procedure, and anticoagulation management will strictly follow established guidelines for discontinuation or bridging. Pre-procedural ultrasound examination will be performed to evaluate cervical vascular anatomy, identify the positions of the common carotid artery, vertebral artery, and internal jugular vein, and rule out contraindications such as anatomical variations, severe vascular tortuosity, or cervical masses.
(2) Intra-procedural monitoring: Throughout the block procedure, patients will be continuously monitored with electrocardiography, non-invasive blood pressure, and pulse oximetry. An intravenous line will be maintained, and emergency medications (atropine, dopamine, lipid emulsion, etc.) will be readily available. A three-step aspiration-injection-aspiration technique will be employed: after needle placement, aspiration is performed to confirm no blood or cerebrospinal fluid; 1 mL of normal saline is injected to assess resistance; then local anesthetic is injected slowly in divided doses, with re-aspiration after each 1 mL injection to prevent intravascular or intrathecal injection.
(3) Adverse event monitoring: An independent DSMB has been established for this study, consisting of experts in anesthesiology, neurology, and biostatistics. The DSMB will oversee the safety of the entire study, regularly review adverse event data, and have the authority to recommend study suspension or termination if safety concerns arise.

#### 3.5.3 Post-block monitoring protocol

After the procedure, patients will be transferred to the post-anesthesia care unit (PACU) or ward and monitored for at least 2 h with continuous vital sign assessment. Key monitoring observations include: voice quality, swallowing function, respiratory rate, chest wall excursion (to rule out pneumothorax), neck swelling (to rule out hematoma), and upper limb sensory/motor function (to rule out brachial plexus involvement).

If hoarseness or dysphagia occurs, patients will be placed in a semi-recumbent position to prevent aspiration, and otolaryngology consultation will be arranged if necessary. Patients with no abnormalities after 2 h may be transferred to the general ward, where ward nurses will continue monitoring according to the nursing record sheet for up to 24 h post-procedure.

#### 3.5.4 Anticoagulation management

The following perioperative anticoagulation management protocol will be followed: Aspirin (oral): Discontinue 7 days before the procedure. Clopidogrel: Discontinue 7 days before the procedure. Warfarin: Discontinue 3–5 days before the procedure; recheck INR before the procedure, target INR < 1.5. Low molecular weight heparin: Discontinue 24 h before the procedure.

For special cases, consultations with cardiology and vascular surgery will be arranged to develop individualized bridging protocols. The block will be performed only after confirming no contraindications to anticoagulation.

#### 3.5.5 Emergency response for local anesthetic systemic toxicity (LAST)

If early symptoms of LAST (perioral numbness, tinnitus, agitation, tachycardia) occur, immediate actions include: stop injection, administer oxygen, and maintain airway patency.

If seizures or convulsions develop, administer propofol or midazolam intravenously to control seizures, and perform endotracheal intubation if necessary.

If circulatory depression occurs, administer intravenous epinephrine and fluid resuscitation to maintain blood pressure.

Administer 20% lipid emulsion according to standard protocol: initial bolus of 1.5 mL/kg, followed by continuous infusion at 0.25 mL/kg/min. Continuous monitoring of electrocardiography, blood pressure, and oxygen saturation is required, and intensive care unit consultation should be arranged if necessary.

#### 3.5.6 Systematic adverse event reporting

Mild adverse events (e.g., transient hoarseness, mild dysphagia, local pain) will be recorded by the operating physician within 24 h using an adverse event reporting form and reported to the study coordinator.

Serious adverse events (e.g., LAST, severe hematoma, pneumothorax, nerve injury, death) will be reported to the ethics committee, DSMB, and institutional adverse event management department within 24 h of discovery.

All adverse events will be documented with details on time of occurrence, severity, management measures, and outcomes. Regular summary reports will be submitted to the DSMB for review.

Stopping rule: If two or more serious adverse events directly related to SGB occur, the DSMB may recommend early termination of the study.

### 3.6 Standardized perioperative management

To ensure that any observed intergroup differences are primarily attributable to the study intervention (SGB or sham block), all participants receive a strictly standardized perioperative care protocol encompassing anesthesia, analgesia, and delirium prevention strategies.

#### 3.6.1 Standardized anesthesia protocol

Anesthesia is managed according to a uniform protocol for induction, maintenance, and emergence.

(1) Preoperative management: Adherence to strict fasting guidelines (8 h for solids, 2 h for clear liquids). Premedication with benzodiazepines is avoided.
(2) Induction: Following standard monitoring establishment, anesthesia is induced with intravenous sufentanil, ciprofol, and cisatracurium. The exact doses are determined as follows: Sufentanil: 0.3 μg/kg for ASA III or age ≥75 years; 0.5 μg/kg for ASA I-II and age <75 years. Propofol: 0.4 mg/kg for age ≥75 years or ASA III; 0.5 mg/kg for age <75 years and ASA I-II. Cisatracurium: 0.15 mg/kg for age ≥75 years or ASA III; 0.2 mg/kg for age <75 years and ASA I-II.
(3) Maintenance: Anesthesia is maintained with sevoflurane (end-tidal concentration 1.0–1.3 MAC, age-adjusted) and a continuous remifentanil infusion. Remifentanil infusion rate is initiated at 0.10 μg/kg/min and adjusted within the range of 0.05–0.20 μg/kg/min to maintain heart rate and blood pressure within ±20% of baseline. Higher rates (0.15–0.20 μg/kg/min) are used for patients <70 years or those with a history of chronic opioid use; lower rates (0.05–0.10 μg/kg/min) are used for patients ≥75 years or those with baseline bradycardia. Supplemental boluses of sufentanil (5–10 μg) are administered if blood pressure or heart rate increases by >20% from baseline. Muscle relaxation is maintained with intermittent cisatracurium as needed. Ventilation is controlled to maintain end-tidal carbon dioxide between 35 and 45 mmHg.
(4) Hemodynamic management: Mean arterial pressure (MAP) is maintained within ±20% of baseline. A decrease in MAP >20% is primarily treated with intravenous ephedrine (6 mg bolus). An increase in MAP >20% is managed by deepening anesthesia (increasing sevoflurane concentration or administering supplemental sufentanil).
(5) Emergence: Approximately 30 min before the anticipated end of surgery, ketorolac tromethamine (30 mg IV) is administered, and the remifentanil infusion is discontinued. Extubation is performed upon meeting standard criteria (consciousness, adequate spontaneous respiration, SpO₂ > 95% on room air).

#### 3.6.2 Standardized postoperative analgesia

After extubation, all patients are connected to a standardized patient-controlled intravenous analgesia (PCIA) pump. Crucially, this analgesia protocol is applied identically to both the SGB and sham groups. The PCIA solution contains hydromorphone (0.15–0.2 mg/kg), nalbuphine (1.0–1.5 mg/kg), and ondansetron (16 mg), diluted to 100 mL with normal saline. Doses are calculated based on ideal body weight: Hydromorphone: 0.15 mg/kg for age ≥75 years or eGFR <60 mL/min; 0.2 mg/kg for age <75 years and normal renal function. Nalbuphine: 1.0 mg/kg for age ≥75 years; 1.5 mg/kg for age <75 years. Ondansetron: 16 mg (fixed dose, independent of body weight). The pump is set to a basal infusion of 1.0 mL/h, a bolus dose of 2.0 mL, a lockout interval of 15 min, and a 1-h limit of 8.0 mL. Rescue analgesia (hydromorphone 0.2–0.4 mg IV) is provided by the acute pain service team if pain is inadequately controlled (NRS ≥ 4) by the PCIA. Total opioid consumption (in morphine milligram equivalents, MME) will be recorded for the first 48 h postoperatively and compared between groups as a secondary outcome.

#### 3.6.3 Multicomponent delirium prevention bundle

All patients receive a non-pharmacologic, multicomponent delirium prevention strategy throughout their hospitalization, adapted from the ABCDEF bundle:

Assess, prevent, and manage pain.

Both spontaneous awakening and breathing trials (when applicable).

Choice of analgesia and sedation.

Delirium: assess, prevent, and manage.

Early mobility and exercise.

Family engagement and empowerment.

Specific interventions include regular reorientation, provision of sensory aids (glasses, hearing aids), encouragement of early mobilization (beginning on postoperative day 1), sleep hygiene promotion (minimizing nocturnal disturbances), and active involvement of family members in care.

To ensure protocol adherence, a daily compliance checklist is developed, recording the completion of each component (reorientation, sensory aids, early mobilization, sleep hygiene, family involvement). Adherence rates will be reported and compared between groups.

#### 3.6.4 Control of confounding variables

To minimize the potential impact of confounding variables on the primary outcome (POD incidence), the following measures will be implemented:

(1) Standardized perioperative management: As described in sections 3.5.1–3.5.3, all patients (both groups) will receive identical anesthesia, analgesia, and delirium prevention protocols. Any deviation from these protocols will be recorded as a protocol deviation.
(2) Data collection on potential confounders: The following predefined variables, known to influence POD risk, will be collected and adjusted for in the statistical analysis: age, sex, body mass index (BMI), ASA physical status, baseline MoCA score, duration of surgery, intraoperative blood loss, intraoperative use of vasopressors, and postoperative opioid consumption (MME).
(3) Statistical adjustment: In addition to the primary intention-to-treat analysis, a multivariable logistic regression model will be used to adjust for the above confounders, reporting adjusted odds ratios (aOR) with 95% confidence intervals.
(4) Sensitivity analysis: A sensitivity analysis excluding patients with major protocol deviations (e.g., those who did not receive the allocated block, or those with incomplete primary outcome data) will be performed to assess the robustness of the findings.

### 3.7 Outcome measures

A summary of the assessment schedule is provided in Table 1 (SPIRIT).

**Table 1:** SPIRIT schedule of enrolment, interventions, and assessments (23).

Study period	Enrolment	Intervention		Postoperative period (daily: POD1-3)	Close-out
Timepoint	≤7 d pre-op	Day-1 p.m.	Day 0	POD1-3	D/C
Enrolment					
Eligibility screen	X				
Informed consent	X				
Interventions					
SGB/Sham block		X	X		
Surgery and standardized care			X		
Data collection					
Demographics, medical history	X				
MoCA	X				
ASA physical status	X				
Outcome measures					
Primary outcome					
POD incidence (3D-CAM)				X (POD1-3 bid)^a^	
Secondary outcomes					
Delirium severity (MDAS)^b^				X (POD1-3 bid)^b^	X (record)
Quality of recovery-15 (QoR-15)				X (on POD3)	
Inflammatory biomarkers (procalcitonin, CRP, IL-6, LMR, NLR)^c^	X (blood)			X (on POD1 and POD3)^c^	
Postop pain intensity (NRS)^d^				X (q8h, POD1-2)^d^	
Opioid consumption (MME)^d^				X (total, POD1-2)^d^	
Postop complications (Clavien-Dindo)^e^				X (daily)^e^	X (record)
Length of hospital stay (days)					X
Safety monitoring					
Adverse events (AEs)		X	X	X (daily)	X

a POD incidence (3D-CAM): assessed twice daily (bid) from postoperative day 1 to day 3.

b Delirium severity score: assessed daily (POD1-3). Total delirium duration is recorded at discharge (D/C).

c Inflammatory biomarkers: blood samples collected at baseline (V0), postoperative day 1, and postoperative day 3.

d Postop pain and opioid use: pain intensity (NRS) is assessed every 8 h (q8h) for the first 48 h only (POD1-2). Total opioid consumption (MME) is calculated and recorded for the first 48 h (POD1-2).

e Postop complications: monitored daily (POD1-7) and recorded at discharge.

D/C, discharge; SGB, stellate ganglion block; MoCA, Montreal Cognitive Assessment; ASA, American Society of Anesthesiologists; POD, postoperative day; 3D-CAM, 3-Minute Diagnostic Interview for CAM-defined Delirium; QoR-15, quality of recovery-15 scale; CRP, C-reactive protein; IL-6, interleukin-6; NRS, numerical rating scale; MME, morphine milligram equivalents; bid, twice daily; q8h, every 8 h.

#### 3.7.1 Primary outcome

Cumulative incidence of POD from postoperative day 1 to day 3, assessed twice daily (morning 8:00–10:00, evening 18:00–20:00) using the validated 3-Minute Diagnostic Interview for CAM-defined Delirium (3D-CAM) (16). Assessors will complete standardized training (including 3D-CAM and MDAS operation, case simulation, and inter-rater reliability testing, target *κ* ≥ 0.8) before formal assessment. For sedated or intubated patients, assessment will be deferred until the patient is awake, alert, and able to cooperate; if unable to evaluate within 24 h, POD status will be recorded as missing. Delirium severity will be further evaluated using the Memorial Delirium Assessment Scale (MDAS) as described in section 3.7.1.1(4) and 3.7.2.

##### 3.7.1.1 Operational details for postoperative delirium assessment

(1) Assessment team and training: Delirium assessments will be performed by two trained research assistants who are blinded to group allocation. Both assistants will undergo a standardized training program consisting of: (1) a 2-h didactic session covering the diagnostic criteria of delirium (DSM-5), the 3D-CAM, and the MDAS; (2) supervised assessments of five pilot patients (not enrolled in the trial) to achieve competency; and (3) an inter-rater reliability test, requiring a kappa coefficient ≥0.80 before starting the formal assessment. Inter-rater reliability will be rechecked every 3 months.
(2) Assessment time points: The 3D-CAM and MDAS will be administered twice daily from postoperative day 1 to day 3, specifically between 8:00–10:00 a.m and 18:00–20:00 p.m. An additional assessment will be performed if a patient shows sudden changes in mental status or behavioral disturbances. At discharge, total duration of delirium (number of days with any positive 3D-CAM assessment) will be recorded.
(3) Assessment for sedated or intubated patients: For patients who are sedated (RASS ≤−2) or intubated, routine 3D-CAM assessment will be deferred until the patient is awake and able to respond. In such cases, the medical record will be reviewed daily for documentation of delirium-related symptoms using the CAM-ICU (for ICU patients) or notes from the clinical team. These patients will not be excluded from the analysis; instead, the reason for missing assessment (e.g., sedation, intubation) will be recorded as a protocol deviation.
(4) Delirium severity assessment: Delirium severity will be assessed using the Memorial Delirium Assessment Scale (MDAS), a 10-item, clinician-rated scale with a total score ranging from 0 to 30 (higher scores indicate more severe delirium). The MDAS will be administered twice daily from postoperative day 1 to day 3, synchronized with the 3D-CAM assessments, conducted immediately after the 3D-CAM is completed, and performed by the same trained research assistants who administer the 3D-CAM.

#### 3.7.2 Secondary outcomes

(1) Delirium severity, assessed using the Memorial Delirium Assessment Scale (MDAS), and total delirium duration (number of days with a positive 3D-CAM assessment).
(2) Quality of recovery- 15 (QoR-15) score on postoperative day 3.
(3) Plasma levels of inflammatory biomarkers (procalcitonin, C-reactive protein, interleukin-6, lymphocyte-to-monocyte ratio [LMR], neutrophil-to-lymphocyte ratio [NLR]) at baseline, postoperative day 1, and postoperative day 3.
(4) Postoperative pain intensity (NRS) trajectories and total opioid consumption (morphine milligram equivalents) over 48 h.
(5) Incidence of postoperative complications (Clavien-Dindo classification) and length of hospital stay.

#### 3.7.3 Safety outcomes

All adverse events (AEs), particularly SGB-related (e.g., hoarseness, dysphagia, hematoma, local anesthetic systemic toxicity) and opioid-related AEs (e.g., respiratory depression, nausea/vomiting), will be recorded.

### 3.8 Sample size calculation

Based on prior literature reporting POD incidences ranging from 35 to 48% (2–4), we adopted a best-case scenario assumption for sample size planning: a baseline incidence of 40% in the sham group and a 50% relative risk reduction (to 20%) in the SGB group. Under these assumptions (*α* = 0.05, power = 80%, two-sided Chi-square test), 78 patients per group are required. Accounting for an estimated 10% dropout rate, the final sample size is 174 participants (87 per group).

We acknowledge that this effect size may be optimistic, especially given that our protocol excludes patients with preexisting cognitive impairment and includes a multicomponent delirium prevention bundle. If the true effect is smaller, this study may be underpowered for the primary outcome. Therefore, the results should be interpreted as hypothesis-generating, and future meta-analyses will be needed to confirm smaller effects.

### 3.9 Statistical analysis

#### 3.9.1 Analysis sets

The primary analysis will follow the intention-to-treat (ITT) principle, including all randomized participants in their originally assigned groups. A per-protocol (PP) population will also be defined for sensitivity analysis, excluding participants with major protocol deviations (e.g., failure to receive the assigned block, surgery duration outside predefined limits, or missing all primary outcome assessments).

#### 3.9.2 Analysis of primary outcomes

The cumulative incidence of POD on postoperative days 1–3 will be compared between groups using the Chi-square test, and relative risk (RR) with a 95% confidence interval (CI) will be reported. A supplementary multivariable logistic regression will be performed to adjust for pre-specified covariates: age, baseline MoCA score, and ASA physical status. To obtain adjusted relative risk instead of odds ratio, modified Poisson regression with robust standard errors will be adopted for covariate-adjusted analysis. Multivariable logistic regression will be used only as a supplementary approach when convergence issues occur.

#### 3.9.3 Analysis of secondary outcomes

Continuous secondary outcomes measured at a single time point (e.g., total QoR-15 score on postoperative day 3, length of hospital stay) will be compared between groups using independent Student’s t-tests if data are normally distributed, or Mann–Whitney *U* tests if the normality assumption is violated. Normality will be assessed using the Shapiro–Wilk test and homoscedasticity using Levene’s test.

For longitudinal data collected at multiple time points (e.g., serial pain NRS scores every 8 h for 48 h, inflammatory biomarkers on POD1 and POD3), we will employ linear mixed-effects models with a random intercept for each patient to account for within-subject correlation. Fixed effects will include treatment group, time, and their interaction term. A significant interaction term would indicate different trajectories of change between groups.

Total opioid consumption over the first 48 postoperative hours (in morphine milligram equivalents, MME) is a count variable and will be analyzed using Poisson regression or negative binomial regression if overdispersion is present. Results will be expressed as incidence rate ratios (IRRs) with 95% confidence intervals. Overdispersion will be verified via Pearson chi-square divided by degrees of freedom. Baseline pain score, operation duration and body weight will be adjusted in regression models. Model fitness will be evaluated by AIC and deviance.

#### 3.9.4 Handling of missing data

Missing data will be addressed using multiple imputation by chained equations (MICE). We will generate *m* = 50 imputed datasets, including the intervention group, primary outcome, and all pre-specified covariates in the imputation model. Pooled estimates will be obtained using Rubin’s rules. Missing POD assessments are assumed to be missing at random. Longitudinal time-point information will be included in the imputation model. Complete-case analysis will be conducted as sensitivity analysis to verify the robustness of primary results.

#### 3.9.5 Statistical significance and software

A two-sided *p*-value <0.05 will be considered significant for the primary outcome. The false discovery rate (FDR) for secondary outcomes will be controlled using the Benjamini-Hochberg procedure. Analyses will be performed using SPSS 26.0 and R 4.3.2.

#### 3.9.6 Interim analysis and stopping guidelines

We have planned a single pre-specified interim analysis to evaluate both efficacy and safety, to potentially stop the trial early for overwhelming benefit, futility, or safety concerns. The details are as follows:

(1) Timing: The interim analysis will be performed after 50% of the target enrollment (approximately 87 patients) have been recruited and have completed the primary outcome assessment (i.e., POD assessment on postoperative days 1–3).
(2) Execution: The analysis will be conducted by the independent Data Safety and Monitoring Board (DSMB). The research team (including investigators and statisticians) will remain blinded until the DSMB communicates its recommendations. All unblinded interim statistical calculations are performed by an independent external statistician, and only summarized results are submitted to DSMB without disclosing group information to the research team.
(3) Statistical boundaries: The O’Brien-Fleming spending function will be used to control the overall two-sided type I error rate at *α* = 0.05. This pre-specified sequential design results in a conservative boundary for the first interim look, requiring stronger evidence for early stopping.

(a) Efficacy boundary (*α*₁): The nominal two-sided significance level for the interim analysis at 50% information is *α*_1_ = 0.003. If the between-group comparison of the primary outcome (cumulative incidence of POD) yields a two-sided *p*-value <0.003, and the safety profile is acceptable, the DSMB may recommend early termination for overwhelming efficacy.
(b) Futility boundary: A non-binding futility boundary will also be evaluated. If the conditional power to achieve a statistically significant result (*p* < 0.05) at the final analysis, given the observed data at interim, is calculated to be less than 10%, the DSMB may recommend early termination for futility. This indicates a very low probability that completing the trial would demonstrate the hypothesized treatment effect.

### 3.10 Data management and monitoring

#### 3.10.1 Data collection

All study data will be collected and managed using the ResMan electronic data capture platform. Data entry will undergo double verification.

Participants will be assigned a unique study identifier (UID); all stored data will be pseudonymized. Access to the final dataset will be restricted to the principal investigator and statistician.

#### 3.10.2 Safety monitoring and DSMB

##### 3.10.2.1 DSMB composition

An independent Data Safety Monitoring Board (DSMB) has been established for this study, consisting of three external independent experts with no affiliation, academic collaboration, or financial relationship with the study team. One senior anesthesiologist from a provincial tertiary hospital, specializing in complication prevention for nerve blocks. One senior neurologist from a provincial tertiary hospital, responsible for assessing nerve injury-related adverse events. One biostatistician specializing in clinical epidemiology, responsible for interim data analysis and risk assessment. None of the DSMB members will participate in participant recruitment, clinical procedures, outcome assessment, or routine data management. All DSMB members will sign a Conflict of Interest Disclosure and a Confidentiality Agreement, committing to independent monitoring throughout the study, and will not disclose group allocation, participant data, or monitoring opinions until study completion and publication. The DSMB term is 2 years, renewable once. Any breach of confidentiality will result in immediate termination of DSMB membership and appropriate accountability.

##### 3.10.2.2 Independence assurance

The DSMB is completely independent of the study implementation team and the sponsor, with independent authority to monitor, evaluate, and recommend study termination. The DSMB’s operational budget is managed independently without interference from the research group. The study team may attend DSMB meetings only to answer questions and may not participate in voting or influence safety decisions, ensuring objective and impartial monitoring.

##### 3.10.2.3 Access to unblinded data

The DSMB will have access only to anonymized unblinded safety data, including participant group allocation, block procedure records, adverse event reports, and baseline medical history and medication data. The DSMB will not have access to personally identifiable information or to primary outcome efficacy statistics before the final analysis, balancing safety monitoring with blind maintenance.

##### 3.10.2.4 Safety review frequency

A dual-mode review system will be implemented: routine scheduled reviews and emergency ad-hoc reviews.

(1) Routine reviews

- (a) Initial review: After the first 10 participants have completed the SGB procedure and 24-h post-procedure monitoring, the DSMB will conduct an initial safety review, focusing on the safety of the SGB procedure, adverse event profiles, and feasibility of the monitoring protocol.
- (b) Regular reviews: After every 20 enrolled participants, the DSMB will conduct a regular safety review, summarizing adverse event data and procedure-related complications, assessing whether safety meets prespecified standards, and reviewing data integrity and completeness.
- (c) Interim review: When enrollment reaches 50% of the total sample size (approximately 87 participants), the DSMB will conduct an interim safety review, integrating results from the independent unblinded interim analysis to assess both safety and efficacy, and decide whether to continue, modify, or terminate the study.
- (d) Final review: After all participants have completed follow-up, the DSMB will conduct a final safety review, summarizing adverse event data across the entire study period, assessing overall safety, and issuing a final DSMB report.

(2) Emergency reviews

- (a) If one SGB-related serious adverse event (e.g., LAST, pneumothorax, severe hematoma, nerve injury) occurs, the study coordinator will submit detailed information to the DSMB within 24 h. The DSMB will immediately initiate an emergency review to assess the association with the intervention and recommend whether to pause the study.
- (b) In cases of public health emergencies, major protocol amendments, or other urgent situations that may affect participant safety, the DSMB may independently initiate an emergency review.

(3) Review format

DSMB meetings will be conducted online or in person, with all members participating. The study coordinator will submit relevant data, and the principal investigator may attend only to answer questions (without voting rights). Written monitoring records will be signed by all DSMB members and archived.

##### 3.10.2.5 Independent unblinded interim analysis

To assess safety and efficacy in a timely manner, an interim analysis will be performed when enrollment reaches 50% of the total sample size (approximately 87 participants), conducted by an independent unblinded statistician.

(1) Statistician qualifications: An independent statistician with no conflict of interest with the study team and with experience in clinical trial statistics will be appointed. This statistician will not participate in any clinical procedures or data collection and will be responsible solely for the design and execution of the interim analysis.
(2) Analysis content: The analysis will focus on comparing adverse event rates (including mild and serious AEs) and SGB-related complication rates between groups, while also providing a preliminary analysis of the primary outcome (cumulative incidence of POD on postoperative days 1–3) to assess safety and efficacy trends.
(3) Unblinding requirements: The independent unblinded statistician will have access to complete unblinded data (including group allocation, adverse event data, and outcome data) and will use prespecified statistical methods (chi-square test, logistic regression, etc.) to conduct the analysis, producing an interim analysis report for the DSMB.
(4) Blind maintenance: During the interim analysis, the independent unblinded statistician will strictly maintain confidentiality, submitting the analysis report only to the DSMB. The study team (all researchers except the SGB operators) will remain blinded throughout, not accessing interim analysis results to avoid compromising objectivity.
(5) DSMB decision-making: The DSMB will review the interim analysis report, focusing primarily on safety. If unacceptable risks are identified, the DSMB may recommend termination. If safety is satisfactory, the DSMB may recommend continuation or, based on interim results, recommend sample size adjustment.

##### 3.10.2.6 Stopping rules for SGB-related adverse events

The study may be terminated if any of the following criteria are met:

Two or more SGB-related serious adverse events occur, including LAST, severe cervical hematoma, pneumothorax, persistent recurrent laryngeal nerve palsy, or intrathecal drug spread. The incidence of serious adverse events in the SGB group exceeds the pre-specified safety threshold of 5%, and the DSMB determines that this risk cannot be mitigated by modifying procedures or enhancing monitoring. The interim analysis shows a significantly higher total adverse event rate in the SGB group compared to the sham group (*p* < 0.05), and the difference is clinically meaningful, indicating unacceptable safety risks. A major safety hazard is identified in the SGB procedure that cannot be corrected through protocol amendments. Data fabrication or serious protocol violations occur that compromise data integrity and cannot be rectified.

If any of the above criteria are met, the DSMB will issue a written recommendation to suspend enrollment, modify the protocol, or terminate the trial. After the DSMB recommends termination, a written report will be submitted to the ethics committee and the Chinese Clinical Trial Registry. The study team will immediately stop participant recruitment and intervention, complete follow-up of enrolled participants to ensure their safety, and report to relevant authorities as required.

### 3.11 Ethics and dissemination

The study protocol (Version 1.0, 2025.12.04) has been approved by the ethics committee of the Second Hospital & Clinical Medical School, Lanzhou University (Approval No. 2025A-1323). Written informed consent will be obtained from all participants. Important protocol modifications will be communicated to the ethics committee, trial registry, and investigators. Results will be disseminated through peer- reviewed scientific journals and conference presentations, regardless of outcome.

## 4 Discussion

This protocol describes the design of a randomized controlled trial to evaluate the efficacy of a single preoperative SGB for preventing POD in gastrointestinal cancer surgery patients (16, 17). The study’s primary strength lies in its rigorous methodology— incorporating randomization, double-blinding with a credible sham procedure, standardized co-interventions, and validated outcome measures—which minimizes bias and enhances the validity of future findings.

The rationale for SGB is grounded in its potential to modulate two key POD pathways: neuroinflammation and autonomic dysregulation (17–21). It is important to emphasize that this rationale is primarily derived from studies on POCD, pain, inflammation, and other perioperative outcomes, rather than from direct evidence on POD (16, 17). Currently, direct clinical evidence for SGB preventing POD remains scarce, with existing human studies being small in scale or still at the protocol stage. Therefore, if our hypothesis is confirmed, SGB could represent a novel, targeted, and non-pharmacologic adjunct to existing delirium-prevention bundles. Its relatively short procedure time and favorable safety profile may facilitate clinical translation.

Several methodological limitations should be acknowledged. First, the single-center design may limit generalizability, though it ensures protocol consistency. Second, while we measure systemic inflammatory markers, direct assessment of central neuroinflammatory changes is not feasible in this clinical setting. Third, our sample size calculation assumed a 50% relative risk reduction, which may be optimistic in an elderly population (≥60 years) that already excludes cognitively impaired patients and additionally receives a standardized multicomponent delirium prevention bundle. Therefore, if the true effect is more modest, the study may be underpowered. The findings should be considered exploratory. Fourth, the biological rationale for SGB preventing POD is largely derived from indirect evidence (studies on POCD, pain, inflammation, and autonomic function). Direct evidence specifically targeting POD remains limited. Positive results would require replication in larger, independent cohorts. Fifth, all SGB procedures are performed on the right side, which limits generalizability to left-sided SGB. Sixth, maintaining blinding is challenging because Horner’s syndrome may be visible to participants and potentially to assessors. Although the performing anesthesiologist is unblinded by necessity and strict role separation (performer vs. assessor) mitigates this risk, we have incorporated a blinding effectiveness assessment (participant and assessor checks, see section 3.3). Results will be reported to allow readers to evaluate blinding success.

This trial will contribute high-level evidence regarding the role of neuromodulation in perioperative brain health. If SGB proves effective, future research could explore optimal dosing (volume/concentration), timing (pre- vs. post-operative), and its effects on long-term cognitive function.
